# Association between childhood trauma and mental health disorders in adolescents during the second pandemic wave of COVID-19, Chiclayo-Peru

**DOI:** 10.3389/fpsyt.2023.1169247

**Published:** 2023-06-22

**Authors:** Mario J. Valladares-Garrido, Darwin A. León-Figueroa, Franccesca M. Dawson, Stefany C. Burga-Cachay, Maria A. Fernandez-Canani, Virgilio E. Failoc-Rojas, César Johan Pereira-Victorio, Danai Valladares-Garrido, Fiorella Inga-Berrospi

**Affiliations:** ^1^Universidad Peruana Cayetano Heredia, Lima, Peru; ^2^Facultad de Medicina Humana, Universidad de San Martín de Porres, Chiclayo, Peru; ^3^Centro de Investigación en Atención Primaria de Salud, Universidad Peruana Cayetano Heredia, Lima, Peru; ^4^Escuela de Medicina, Universidad Privada Antenor Orrego, Piura, Peru; ^5^Research Unit for Generation and Synthesis Evidence in Health, Universidad San Ignacio de Loyola, Lima, Peru; ^6^School of Medicine, Universidad Continental, Lima, Peru; ^7^Escuela de Medicina, Universidad Cesar Vallejo, Piura, Peru; ^8^Unidad de Epidemiología y Salud Ambiental, Hospital de Apoyo II Santa Rosa, Piura, Peru; ^9^Grupo de Investigación en Gestión y Salud Pública, Universidad Norbert Wiener, Lima, Peru

**Keywords:** childhood trauma, mental health, depression, anxiety, adolescents, COVID-19, pandemic, Peru

## Abstract

**Introduction:**

The COVID-19 pandemic has significantly affected mental health, with children and adolescents being particularly vulnerable. Evidence on the association between childhood trauma and mental health outcomes in schoolchildren during the pandemic is limited. This study aimed to evaluate this relationship in Chiclayo city, northern Peru, during the second wave of COVID-19.

**Methods:**

A cross-sectional secondary data study was conducted, measuring childhood trauma using the Marshall’s Trauma Scale, depressive symptomatology (PHQ-9), and anxiety symptomatology (GAD-7). Additional variables assessed were alcohol use (AUDIT), resilience (abbreviated CD-RISC), and socio-educational data. Prevalence ratios were estimated using generalized linear models.

**Results:**

Among 456 participants, 88.2% were female, with a mean age of 14.5 years (SD: 1.33). Depressive symptomatology prevalence was 76.3% (95%CI: 72.14–80.15) and increased by 23% in schoolchildren with childhood trauma (PR: 1.23; 95%CI: 1.10–1.37). Factors positively associated with depressive symptomatology included increasing age, seeking mental health help during the pandemic, and severe family dysfunction. Anxiety symptomatology prevalence was 62.3% (95%CI: 57.65–66.75) and increased by 55% in schoolchildren with childhood trauma (PR: 1.55; 95%CI: 1.31–1.85). Anxiety symptomatology was positively associated with mild, moderate, and severe family dysfunction.

**Conclusion:**

Schoolchildren exposed to childhood trauma are at increased risk for depressive and anxiety symptoms. Monitoring the impact of the COVID-19 pandemic on adolescent mental health is vital. These findings can assist schools in establishing effective measures to prevent mental health outcomes.

## Introduction

According to UNICEF, 1 in 7 children globally has experienced a minimum of 9 months of mandatory or recommended confinement since the beginning of the COVID-19 pandemic (1). Numerous studies have shown that the pandemic has led to a decline in mental health (2–6), with children and adolescents emerging as a particularly vulnerable group (7–10). This can be attributed to the adverse effects of enforced social isolation on young people (11). During the pandemic’s first year, the prevalence of mental health issues in children and adolescents increased by 20%, with depressive and anxious symptoms estimated at 25.2 and 20%, respectively (12). In Peru, a study found that 45.6 and 36.8% of surveyed adolescents reported anxiety and depression symptoms during the first wave of COVID-19, respectively (13). Another study with a smaller sample size noted a greater increase in depressive symptoms among women (14). While multiple research studies have examined the influence of the COVID-19 pandemic on mental well-being during the initial wave (15–17), research on the second wave is limited, despite the increasing likelihood of developing mental illnesses with prolonged isolation (18).

Research has shown that the development of mental illnesses such as anxiety and post-traumatic stress disorder can be rooted in exposure to fear, like the fear of losing a loved one experienced during the COVID-19 pandemic (19, 20). Furthermore, having a family member infected with COVID-19, media overload, being in the final year of school, a history of mental illness, and a history of eating disorders prior to the pandemic (21) are factors influencing mental health outcomes in adolescents (22). The heightened prevalence of depressive, anxiety, and post-traumatic stress symptoms in children and adolescents (23) during the COVID-19 pandemic has been associated with factors such as sleep duration, hours dedicated to schoolwork (24), physical activity, having parents working in healthcare professions (25), and dependence on technology and the internet (26). However, there is limited evidence on the influence of childhood trauma on mental health during the pandemic, particularly among the adolescent population.

Childhood trauma has been identified as a significant risk factor for developing mental health disorders later in life and has been associated with increased vulnerability to the negative impacts of stress and adversity (27). Previous studies have indicated that childhood trauma plays a role in the development of an unfavorable mental state during the COVID-19 pandemic (28, 29). Trauma at an early age can cause an over-activation of the hypothalamus, releasing corticotropin-releasing hormone (CRF), which would lead to an increase in stress hormones such as cortisol and adrenaline (30–33). If these levels remain elevated, they can produce neurobiological changes in the cerebral cortex, predisposing the child to adult psychiatric illnesses (30–33).

Despite a growing body of research on the general impact of the pandemic on mental health (34), few studies have specifically explored the role of childhood trauma in this context. For instance, one study investigated the relationship between childhood trauma and mental health disorders during the pandemic but focused on a small sample size (35). Another study examined the association between childhood maltreatment and mental health outcomes in the context of COVID-19 but was limited to adult participants (36). Furthermore, a study conducted in China evaluated the relationship between adverse childhood experiences and mental health problems during the pandemic but did not specifically focus on adolescents (37). These studies, while informative, highlight a gap in the literature and indicate a critical need for further research in this area to better understand the impact of childhood trauma on mental health during the pandemic, particularly among the adolescent population.

Additionally, the available evidence predominantly originates from countries with strong economies, with limited studies developed in low- and middle-income countries (38, 39). This further underscores the necessity for more context-specific research, as the experiences and challenges faced by adolescents in different socio-cultural contexts may vary considerably.

Moreover, existing studies have primarily focused on the immediate psychological consequences of the pandemic, with a lack of longitudinal research investigating the long-term effects of childhood trauma on mental health outcomes in the context of COVID-19 (40–42). This points to the need for studies that not only examine the short-term consequences but also assess the enduring impact of childhood trauma during the pandemic on adolescent mental health.

The objective of this study is to evaluate whether childhood trauma is associated with mental health disorders in adolescents in the Chiclayo region of Peru during the second pandemic wave of COVID-19. This study aims to contribute to the limited existing literature on the influence of childhood trauma on mental health disorders in adolescents during the COVID-19 pandemic. The study will provide valuable insights into the mental health of adolescents during the pandemic, highlighting the need for appropriate strategies to mitigate the negative consequences of COVID-19 on the mental well-being of the most vulnerable groups.

In this study, the research framework is based on the relationship between childhood trauma, other factors (such as demographics, pandemic-related variables, mental health history, resilience, alcohol use, and family functioning), and the two mental health outcomes of depression and anxiety in schoolchildren. We hypothesized that childhood trauma, along with other factors, would be associated with an increased risk of depression and anxiety in schoolchildren. The study’s research framework assumes that childhood trauma can have long-term impacts on mental health outcomes, and that certain factors can either exacerbate or mitigate these impacts. The framework is also grounded in the idea that mental health outcomes are complex and multifactorial, influenced by a variety of individual and environmental factors (43).

## Methods

### Study design

In this study, a cross-sectional analytic approach utilizing secondary data was employed to investigate the relationship between childhood trauma and mental health disorders amid the COVID-19 pandemic. “Secondary data” denotes information initially gathered for another research objective, which is then reanalyzed for the present study. Specifically, the primary research examined the association between family dysfunction and post-traumatic stress disorder (44) from March to April 2021, involving high school students from three schools in Chiclayo, Peru. The current investigation leverages this pre-existing dataset, repurposing it to analyze the connection between childhood trauma and mental health disorders in the same study population, considering the backdrop of the pandemic.

### Population and sample

The study population included 863 adolescent schoolchildren from three schools in Chiclayo, Peru. For the primary study, the sample size was estimated at 520 participants, considering a 43% prevalence of mental health disorders in unexposed individuals, 57% prevalence in exposed individuals, a 5% margin of error, 80% statistical power, and an additional 30% to account for refusals and incomplete registrations (44). Snowball sampling was employed as a recruitment strategy in the primary study (44).

Inclusion criteria for the primary study encompassed schoolchildren aged 11–18 years. The study population was selected because these children are at an age where they are particularly vulnerable to mental health problems, and schools are a key setting for identifying and addressing these problems (45). Additional inclusion criteria were those whose parents provided consent for their participation, and those who gave their assent to answer the questionnaire. Schoolchildren who did not adequately respond to the Child PTSD Symptom Scale and Family APGAR instrument were excluded, resulting in a dataset of 562 participants.

For this secondary analysis, 106 questionnaires with incomplete data for variables of interest (childhood trauma, depressive symptomatology, and anxiety symptomatology) were excluded, yielding a final analytic sample of 456 records.

### Data collection procedures

First, approval was obtained from the heads of the secondary schools to carry out the study on schoolchildren enrolled in the 2021 academic year. Then, informed consent and online assent were requested from parents and students, respectively. Finally, the questionnaire was disseminated online using the REDCap data entry system, using the educational platform of the three schools and official social network groups of each academic year. The survey was disseminated during days when the students were not taking academic evaluations, and the average time for filling out the questionnaire was 15 min.

### Instruments

The questionnaire used in this study aimed to measure various factors that could be associated with mental health disorders in schoolchildren. It includes questions about sociodemographic characteristics, compliance with isolation measures, severity rating of the COVID-19 pandemic, confidence in the government’s ability to manage the epidemic, history of mental health disorders, seeking mental health support, resilience, alcohol consumption, family APGAR, childhood trauma, and symptomatology related to depression and anxiety. Below, we describe the use of validates scales to measure complex variables included in the questionnaire.

#### Childhood trauma (Marshall’s Trauma Scale)

Childhood trauma was assessed using the Marshall’s Trauma Scale, a questionnaire specifically designed to measure childhood trauma in Peruvian populations. This instrument comprises seven questions (46), with a maximum score of seven points, and is categorized into two groups based on the results: The scoring system involves assigning a value of 1 or more points for the presence of trauma, and 0 points for the absence of trauma. The scale exhibits excellent external validity, with a correlation coefficient of 0.88 (47), and has been utilized and verified in research carried out in Latin America, including Peru (48, 49).

#### Depressive symptomatology (PHQ-9)

This questionnaire consists of 9 questions, each of which is evaluated on a Likert scale from 0 (never) to 1 (some days), 2 (more than half of the days) and 3 (almost every day), with a possible range of total scores from 0 to 27 (50, 51). It is categorized as minimal depression if it receives a score of 0 to 4, mild if it receives a score of 5 to 9, moderate if it receives a score of 10 to 14, moderate–severe if it receives a score of 15 to 19, and severe if it has a score of 20 to 27 (50). In addition, the questionnaire has high psychometric qualities, has been validated in primary care settings with a Latino population and exhibits optimal internal consistency (α = 0.87), as well as adequate convergent and divergent validity and a score equal to or greater than the cut-off point of seven (52, 53). In a sizable sample of the Peruvian population, the study identified adequate invariance when comparing different groups (52).

#### Anxiety symptomatology (GAD-7)

It is an instrument that measures current levels of anxiety symptomatology (54), consisting of 7 questions with a Likert-type rating scale from 0 to 3 (55). The total score ranges from 0 to 21 (56). Scores from zero to four for no anxiety symptoms, five to nine for mild anxiety symptoms, 10 to 14 for moderate anxiety symptoms, and 15 to 21 for severe anxiety symptoms (57). The instrument demonstrates strong internal validity (α = 0.94) when assessed in a Hispanic American sample (58). Employing a cutoff point above 10 points results in excellent sensitivity (97%), specificity (100%), positive predictive value (>97%), and negative predictive value (0.833) in the evaluation (59). The alpha coefficient of the study was 0.93.

#### Alcohol consumption (Alcohol Use Disorders Identification Test – AUDIT)

The World Health Organization (WHO) developed the Alcohol Use Disorders Identification Test (AUDIT) as a rapid screening tool to detect alcohol consumption and identify excessive alcohol intake (60). This ten-question questionnaire, originally created by Saunders (61, 62), was later translated into Spanish and validated by Rubio et al. for primary care patients (63). The instrument exhibited strong internal consistency (α = 0.86). Additionally, when utilizing a specific cut-off point of 8, it achieved substantial sensitivity (90%) and specificity (90%) (63).

The AUDIT questionnaire uses a Likert scale to assess the frequency and severity of alcohol consumption and related problems. The response options for each item vary, but they all use a scale from 0 to 4, where 0 indicates “never,” and 4 indicates “daily or almost daily.” The questionnaire is composed of three sections: three questions about alcohol consumption (quantity and frequency), four questions regarding addiction, and three questions examining the effects (60). The items in the AUDIT questionnaire are (60):

1. How often do you have a drink containing alcohol?
2. How many standard drinks containing alcohol do you have on a typical day when you are drinking?
3. How often do you have six or more drinks on one occasion?
4. How often during the last year have you found that you were not able to stop drinking once you had started?
5. How often during the last year have you failed to do what was normally expected of you because of drinking?
6. How often during the last year have you needed a first drink in the morning to get yourself going after a heavy drinking session?
7. How often during the last year have you had a feeling of guilt or remorse after drinking?
8. How often during the last year have you been unable to remember what happened the night before because you had been drinking?
9. Have you or someone else been injured as a result of your drinking?
10. Has a relative, friend, or healthcare worker been concerned about your drinking or suggested you cut down?

Scores range from 0 to 40, and the questionnaire categorizes risk levels as low (0–7 points), moderate (8–15), high (16–19), and potentially addictive (20 points or more) (64).

#### Resilience (abbreviated CD-RISC)

This survey is made up of 10 questions, each with a Likert scale of 0 to 4 points (65, 66). The instrument has undergone validation in diverse populations, including Spanish-speaking youth, workers from different professions, and healthcare workers (65, 67). Using a cut-off score of 23 or less, the instrument exhibits outstanding psychometric characteristics for discriminating depression among healthcare professionals. It demonstrates strong internal consistency and effectively distinguishes individuals in this population with high sensitivity and specificity (68). The alpha coefficient of this study was 0.95.

### Variables

**Figure 1:**
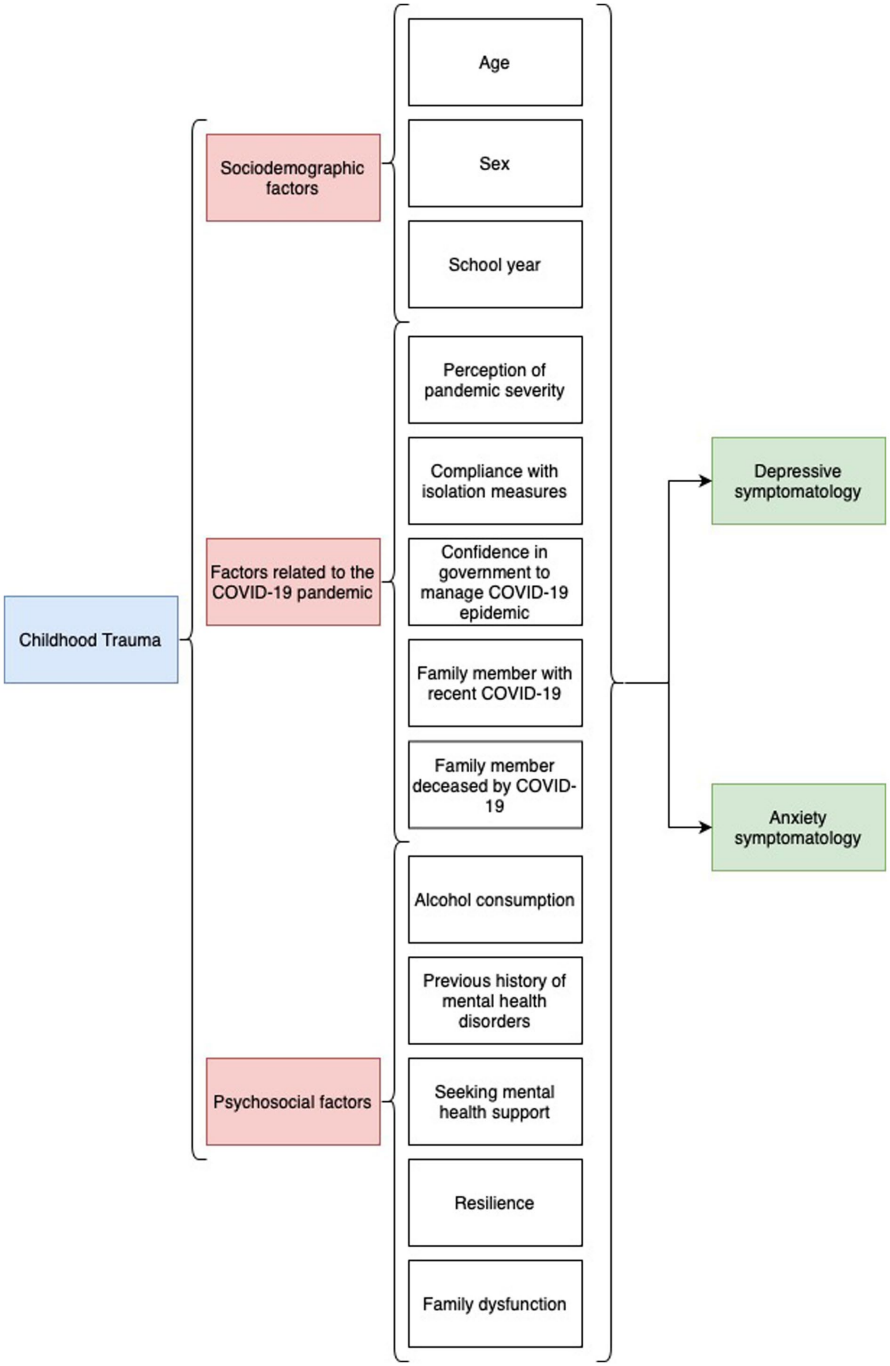
Conceptual model of the study variables and relationships. This figure illustrates the research framework and relationships between the main independent variable, childhood trauma, and the dependent variables, depressive symptomatology, and anxiety symptomatology. It also shows the secondary independent variables (e.g., sex, age, school year, family dysfunction, resilience, alcohol consumption, etc.) and their potential associations with the dependent variables. The arrows represent the direction of influence or interaction among the variables.

To better understand the research framework and relationships between variables, we created a conceptual model (Figure 1). This model illustrates the main independent variable, childhood trauma, and its potential association with the dependent variables, depressive symptomatology, and anxiety symptomatology. Additionally, the model includes the secondary independent variables (e.g., sex, age, school year, family dysfunction, resilience, alcohol consumption, etc.) and shows how they may potentially affect or interact with the dependent variables.

Dependent variables: Depressive symptomatology (PHQ-9 score > 4) and anxiety symptomatology (GAD-7 score > 4).

Main independent variable: Childhood trauma (Marshall Scale score ≥ 1).

Secondary independent variables: Sex (female, male), age in years, school year (first, second, third, fourth, fifth), family dysfunction (normal, mild, moderate, severe), resilience, alcohol consumption (low, medium, high risk and probable addiction), acceptance of quarantine measures (no, yes), severity of the COVID-19 pandemic (very severe, severe, neutral, overestimated, very overestimated), confidence in government response to COVID-19 epidemic (fairly confident, low confidence, neither confident nor distrustful, low distrustful, fairly distrustful), report of having family member with recent COVID-19 (no, yes), report of having family member died from COVID-19 (no, yes), previous history of mental health problems (no, yes), report of having sought mental health help (no, yes).

### Data analysis

For the purpose of descriptive analysis, categorical variables were summarized by displaying frequencies and percentages. Meanwhile, numerical variables were characterized by providing the mean and standard deviation values, after evaluating normal distribution.

We assessed the association between childhood trauma and depressive/anxiety symptoms through a bivariate analysis utilizing the chi-square test of independence; similarly, this was done for the other categorical independent variables. After assessing the assumption of normal distribution, Student’s *t*-test was effective for the numerical variables.

In the simple and multiple regression analysis, we assessed the strength and magnitude of the association of interest (childhood trauma vs. depressive/anxiety symptoms), using generalized linear models (GLM), Poisson family, robust variance, and log link function. In the simple model, we estimated prevalence ratios (PR) and 95% confidence intervals (95%CI) for both the association of interest (childhood trauma vs. depressive/anxiety symptoms) and the confounding variables. In the multiple model, we controlled for the association of interest (childhood trauma and depressive/anxiety symptoms) with the confounding variables. We assessed collinearity of confounding variables included in the multiple model.

Data were examined with Stata 17.0 (StataCorp LP, College Station, TX, United States).

### Ethical aspects

The Ethics and Research Committee of the Universidad San Martín de Porres, Lima, Peru, approved the study. Informed parental consent was required, and informed assent was obtained from each student. Data confidentiality was always guaranteed, and the database was anonymous.

## Results

### General characteristics

Table 1 presents the characteristics of 456 schoolchildren from three schools in Chiclayo, 2021. The mean age was 14.5 years (±1.33), with 88.2% being female. The academic year distribution was as follows: 11.2% in the first year, 10.8% in the second, 12.9% in the third, 26.8% in the fourth, and 38.4% in the fifth. Most participants (96.5%) complied with isolation measures, and 71.5% rated the COVID-19 pandemic as very serious. Regarding confidence in the government’s management of the COVID-19 epidemic, 3.3% reported having a lot of confidence, 27.9% had some confidence, 32.9% were neutral, 20.6% had some distrust, and 15.4% had a lot of distrust.

**Table 1:** Characteristics of schoolchildren in three schools in Chiclayo, 2021 (*n* = 456).

Characteristics		*N* (%)
Age (years)*		14.5 ± 1.33
Sex		
	Male	54 (11.8)	Female	402 (88.2)
Academic year		
	First	51 (11.2)	Second	49 (10.8)	Third	59 (12.9)	Fourth	122 (26.8)	Fifth	175 (38.4)
Compliance with isolation measures		
	No	16 (3.5)	Yes	440 (96.5)
COVID-19 pandemic severity rating		
	Very serious	326 (71.5)	Serious	87 (19.1)	Neutral	21 (4.6)	Overvalued	9 (2.0)	Highly overvalued	13 (2.9)
Confidence in government to manage COVID-19 epidemic		
	A lot of confidence	15 (3.3)	Some confidence	127 (27.9)	Neither trust nor distrust	150 (32.9)	Some distrust	94 (20.6)	A lot of distrust	70 (15.4)
Family member with recent COVID-19		
	No	115 (25.2)	Yes	341 (74.8)
Family member deceased by COVID-19		
	No	234 (51.3)	Yes	222 (48.7)
Previous history of mental health disorders		
	No	397 (87.1)	Yes	59 (12.9)
Seeking mental health support		
	No	371 (81.4)	Yes	85 (18.6)
Resilience		24.3 ± 8.1
Alcohol consumption		
	Low risk	425 (93.2)	Medium risk	25 (5.5)	High risk	4 (0.9)	Probable addiction	2 (0.4)
Family dysfunction		
	No	151 (33.1)	Mild	113 (24.8)	Moderate	85 (18.6)	Severe	107 (23.5)
Childhood trauma		
	No	263 (57.7)	Yes	193 (42.3)
Depressive symptomatology		
	Minimal	108 (23.7)	Mild	133 (29.2)	Moderate	91 (20.0)	Moderately severe	65 (14.3)	Severe	59 (12.9)
Anxiety symptomatology		
	No	172 (37.7)	Mild	127 (27.9)	Moderate	91 (20.0)	Severe	66 (14.5)

*Mean and standard deviation.

In the sample, 74.8% of the schoolchildren had a family member with recent COVID-19, and 48.7% had a family member who died from the disease. Among them, 12.9% had a history of mental health disorders, and 18.6% sought mental health support. The mean resilience score was 24.3 (±8.1). Alcohol consumption was low risk for 93.2% of the participants, medium risk for 5.5%, high risk for 0.9%, and probable addiction for 0.4%. Family dysfunction was reported as none by 33.1%, mild by 24.8%, moderate by 18.6%, and severe by 23.5% of the students.

Childhood trauma was reported by 42.3% of the schoolchildren. Depressive symptomatology was categorized as minimal for 23.7%, mild for 29.2%, moderate for 20.0%, moderately severe for 14.3%, and severe for 12.9% of the participants. Anxiety levels were reported as no anxiety by 37.7%, mild by 27.9%, moderate by 20.0%, and severe by 14.5% of the schoolchildren.

**Figure 2:**
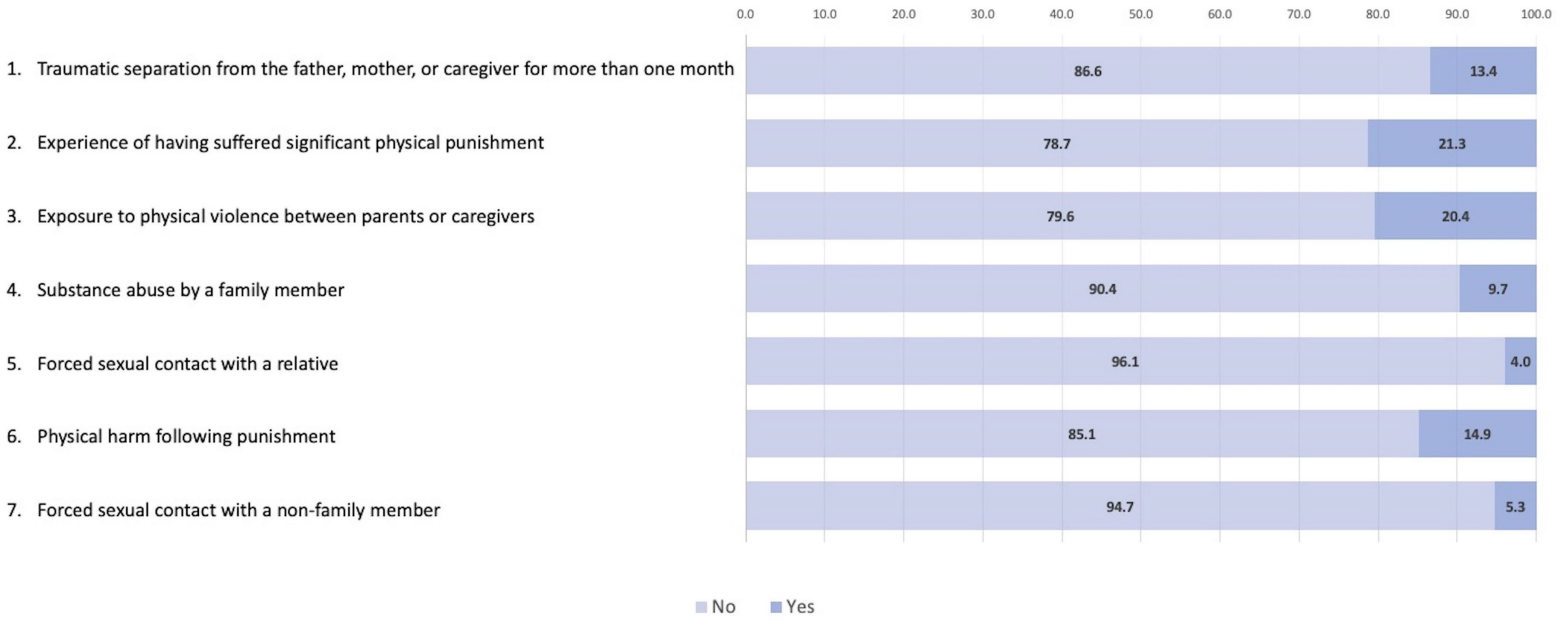
Distribution of responses to Marshall’s Childhood Trauma Questionnaire.

In Figure 2, we present the prevalence of various forms of childhood trauma among the schoolchildren. The most common trauma experienced was significant physical punishment, reported by 21.3% of the participants. The second most frequent trauma was exposure to physical violence between parents or caregivers, which affected 20.4% of the students. Additionally, 13.4% of the schoolchildren faced traumatic separation from a father, mother, or caregiver for more than 1 month, and 14.9% experienced physical harm following punishment. Substance abuse by a family member was reported by 9.7% of the participants. Less common traumas included forced sexual contact with a relative (4.0%) and forced sexual contact with a non-family member (5.3%).

### Bivariate analysis of childhood trauma and mental health outcomes

Childhood trauma was reported by 173 out of 522 participants (33.1%) and was strongly associated with depressive symptomatology and anxiety symptomatology (*p* < 0.001 for both). Those reporting a history of childhood trauma had significantly higher rates of symptoms compared to those without such a history (Table 2).

**Table 2:** Childhood trauma and other factors associated with mental health disorders, in bivariate analysis.

Variables		*Depressive symptomatology*		p*	*Anxiety symptomatology*		p*
		No (*n* = 108)	Yes (*n* = 348)		No (*n* = 172)	Yes (*n* = 284)	
		n (%)	n (%)		n (%)	n (%)	
Age (years)¶**		14.27 ± 1.56	14.64 ± 1.24	0.026	14.29 ± 1.49	14.70 ± 1.20	0.003
Sex				0.076			0.001
	Male	18 (33.3)	36 (66.7)		31 (57.4)	23 (42.6)		Female	90 (22.4)	312 (77.6)		141 (35.1)	261 (64.9)	
Academic year				<0.001			<0.001
	First	23 (45.1)	28 (54.9)		32 (62.8)	19 (37.3)		Second	17 (34.7)	32 (65.3)		24 (49.0)	25 (51.0)		Third	9 (15.3)	50 (84.8)		20 (33.9)	39 (66.1)		Fourth	19 (15.6)	103 (84.4)		39 (32.0)	83 (68.0)		Fifth	40 (22.9)	135 (77.1)		57 (32.6)	118 (67.4)	
Compliance with isolation measures				0.284			0.587
	No	2 (12.5)	14 (87.5)		5 (31.3)	11 (68.8)		Yes	106 (24.1)	334 (75.9)		167 (38.0)	273 (62.1)	
COVID-19 pandemic severity rating				0.222			0.271
	Very serious	85 (26.1)	241 (73.9)		125 (38.3)	201 (61.7)		Serious	16 (18.4)	71 (81.6)		27 (31.0)	60 (69.0)		Neutral	3 (14.3)	18 (85.7)		12 (57.1)	9 (42.9)		Overvalued	3 (33.3)	6 (66.7)		3 (33.3)	6 (66.7)		Highly overvalued	1 (7.7)	12 (92.3)		5 (38.5)	8 (61.5)	
Confidence in government to manage COVID-19 epidemic				0.020			0.255
	A lot of confidence	7 (46.7)	8 (53.3)		8 (53.3)	7 (46.7)		Some confidence	39 (30.7)	88 (69.3)		55 (43.3)	72 (56.7)		Neither trust nor distrust	33 (22.0)	117 (78.0)		56 (37.3)	94 (62.7)		Some distrust	15 (16.0)	79 (84.0)		31 (33.0)	63 (67.0)		A lot of distrust	14 (20.0)	56 (80.0)		22 (31.4)	48 (68.6)	
Family member with recent COVID-19				0.144			0.141
	No	33 (28.7)	82 (71.3)		50 (43.5)	65 (56.5)		Yes	75 (22.0)	266 (78.0)		122 (35.8)	219 (64.2)	
Family member deceased by COVID-19				0.570			0.597
	No	58 (24.8)	176 (75.2)		91 (38.9)	143 (61.1)		Yes	50 (22.5)	172 (77.5)		81 (36.5)	141 (63.5)	
Previous history of mental health disorders				<0.001			<0.001
	No	105 (26.5)	292 (73.6)		166 (41.8)	231 (58.2)		Yes	3 (5.1)	56 (94.9)		6 (10.2)	53 (89.8)	
Seeking mental health support				0.004			0.001
	No	98 (26.4)	273 (73.6)		153 (41.2)	218 (58.8)		Yes	10 (11.8)	75 (88.2)		19 (22.4)	66 (77.7)	
Resilience**		26.6 ± 9.58	23.6 ± 7.41	0.001	26.27 ± 8.88	23.11 ± 7.29	<0.001
Alcohol				0.144			0.072
	Low risk	104 (24.5)	321 (75.5)		165 (38.8)	260 (61.2)		Medium risk-probable addiction	4 (12.9)	27 (87.1)		7 (22.6)	24 (77.4)	
Family Apgar				<0.001			<0.001
	Normal	67 (44.4)	84 (55.6)		85 (56.3)	66 (43.7)		Mild	20 (17.7)	93 (82.3)		44 (38.9)	69 (61.1)		Moderate	5 (5.9)	80 (94.1)		21 (24.7)	64 (75.3)		Severe	16 (15.0)	91 (85.1)		22 (20.6)	85 (79.4)	
Childhood trauma				<0.001			<0.001
	No	88 (33.5)	175 (66.5)		139 (52.9)	124 (47.2)		Yes	20 (10.4)	173 (89.6)		33 (17.1)	160 (82.9)	

*p-value calculated with the Chi-square test for independence.

**Mean and standard deviation.

¶*p*-value calculated with Student’s *t*-test.

Among other variables examined, sex was marginally associated with depressive symptomatology (*p* = 0.076) and significantly associated with anxiety symptomatology (*p* = 0.001), with females reporting higher rates of anxiety symptoms. Academic year was significantly associated with both depressive symptomatology (*p* < 0.001) and anxiety symptomatology (*p* < 0.001), with higher rates of symptoms reported by students in the first and second years compared to those in the third, fourth, and fifth years.

Confidence in the government to manage the COVID-19 epidemic was significantly associated with depressive symptomatology (*p* = 0.020), with those having some or a lot of distrust reporting higher rates of symptoms. Seeking mental health support was significantly associated with both depressive symptomatology (*p* = 0.004) and anxiety symptomatology (*p* = 0.001).

Resilience was found to be significantly associated with both depressive symptomatology (*p* = 0.001) and anxiety symptomatology (*p* < 0.001), with lower levels of resilience associated with higher rates of symptoms.

Other variables examined, including age, compliance with isolation measures, COVID-19 pandemic severity rating, family member with recent COVID-19, family member deceased by COVID-19, previous history of mental health disorders, alcohol use, and family Apgar, did not show significant associations with depressive or anxiety symptomatology in bivariate analysis.

### Simple and multiple regression analyses of childhood trauma and mental health outcomes

Table 3 presents the results of simple and multiple regression analyses examining the association between childhood trauma and mental health disorders in schoolchildren from three schools in Chiclayo, 2021. The factors investigated include age, sex, compliance with isolation measures, COVID-19 pandemic severity rating, confidence in government to manage the COVID-19 epidemic, family member with recent COVID-19, family member deceased by COVID-19, previous history of mental health disorders, seeking mental health support, resilience, alcohol consumption, family Apgar, and childhood trauma.

**Table 3:** Childhood trauma and other factors associated with mental health disorders in schoolchildren from three schools in Chiclayo, 2021, in simple and multiple regression analysis.

Characteristics		*Depressive symptomatology*						*Anxiety symptomatology*					
		Simple regression			Multiple regression*			Simple regression			Multiple regression*		
		PR	95%CI	p**	PR	95CI%	p**	PR	95CI%	p**	PR	95CI%	p**
Age (years)		1.06	0.98–1.14	0.135	1.03	1.01–1.06	0.012	1.09	1.00–1.20	0.053	1.09	1.04–1.13	<0.001
Sex													
	Male	Ref.			Ref.			Ref.			Ref.			Female	1.20	0.90–1.60	0.216	1.11	0.92–1.34	0.255	1.55	1.16–2.09	0.003	1.27	0.98–1.65	0.069
Compliance with isolation measures	
	No	Ref.			Ref.			Ref.			Ref.			Yes	0.86	0.67–1.10	0.236	1.01	0.82–1.25	0.916	0.90	0.67–1.21	0.493	1.09	0.75–1.59	0.650
COVID-19 pandemic severity rating	
	Very serious	Ref.			Ref.			Ref.			Ref.			Serious	1.11	1.01–1.23	0.036	1.07	0.97–1.19	0.161	1.13	0.95–1.34	0.175	1.11	0.93–1.31	0.246	Neutral	1.17	1.00–1.37	0.056	1.08	0.97–1.20	0.148	0.70	0.66–0.73	<0.001	0.64	0.54–0.76	<0.001	Overvalued	0.90	0.60–1.36	0.625	0.79	0.46–1.36	0.399	1.08	0.84–1.40	0.540	0.93	0.66–1.31	0.677	Highly overvalued	1.25	1.11–1.41	<0.001	1.21	0.99–1.49	0.065	1.00	0.58–1.73	0.999	0.95	0.62–1.45	0.809
Confidence in government to manage COVID-19 epidemic	
	A lot of confidence	Ref.			Ref.			Ref.			Ref.			Some confidence	1.38	0.61–3.13	0.435	1.12	0.50–2.50	0.789	1.29	0.62–2.68	0.502	0.96	0.57–1.61	0.875	Neither trust nor distrust	1.56	0.67–3.64	0.302	1.15	0.53–2.50	0.721	1.44	0.68–3.01	0.343	0.95	0.61–1.48	0.806	Some distrust	1.69	0.67–4.23	0.264	1.26	0.55–2.90	0.586	1.54	0.71–3.36	0.278	1.00	0.63–1.60	0.984	A lot of distrust	1.62	0.79–3.32	0.192	1.14	0.56–2.32	0.724	1.57	0.87–2.83	0.136	0.98	0.63–1.53	0.934
Family member with recent COVID-19	
	No	Ref.			Ref.			Ref.			Ref.			Yes	1.12	1.01–1.24	0.029	1.05	0.96–1.15	0.270	1.14	1.06–1.22	<0.001	1.06	0.93–1.21	0.348
Family member deceased by COVID-19	
	No	Ref.			Ref.			Ref.			Ref.			Yes	1.04	0.97–1.11	0.259	1.00	0.96–1.04	0.943	1.04	0.86–1.26	0.679	0.97	0.81–1.16	0.730
Previous history of mental health disorders	
	No	Ref.			Ref.			Ref.			Ref.			Yes	1.29	1.22–1.37	<0.001	1.06	0.92–1.22	0.457	1.54	1.41–1.69	<0.001	1.12	0.91–1.39	0.292
Seeking mental health support													
	No	Ref.			Ref.			Ref.			Ref.			Yes	1.19	1.09–1.29	<0.001	1.12	1.02–1.22	0.016	1.33	1.06–1.66	0.012	1.15	0.88–1.50	0.304
Resilience		0.99	0.98–0.99	<0.001	0.99	0.99–1.00	0.056	0.98	0.97–0.99	<0.001	0.99	0.98–0.99	0.012
Alcohol													
	Low risk	Ref.			Ref.			Ref.			Ref.			Medium risk-probable addiction	1.16	1.03–1.31	0.013	1.01	0.80–1.26	0.957	1.28	1.04–1.58	0.021	1.03	0.80–1.33	0.793
Family Apgar													
	Normal	Ref.			Ref.			Ref.			Ref.			Mild	1.43	1.22–1.67	<0.001	1.42	1.23–1.64	<0.001	1.38	1.05–1.82	0.023	1.30	1.04–1.61	0.020	Moderate	1.65	1.43–1.90	<0.001	1.50	1.35–1.68	<0.001	1.70	1.25–2.32	0.001	1.47	1.16–1.86	0.002	Severe	1.48	1.17–1.87	0.001	1.28	1.01–1.64	0.044	1.80	1.33–2.42	<0.001	1.37	1.05–1.79	0.021
Childhood trauma													
	No	Ref.			Ref.			Ref.			Ref.			Yes	1.35	1.26–1.44	<0.001	1.23	1.10–1.37	<0.001	1.76	1.57–1.98	<0.001	1.55	1.31–1.85	<0.001

*Adjusted for covariates of interest.

**p-values obtained with Generalized Linear Models (GLM), Poisson family, log-link function, robust variance.

In the multiple regression analysis, childhood trauma demonstrated a significant association with depressive symptomatology (PR = 1.23, 95% CI: 1.10–1.37, *p* < 0.001) and anxiety symptomatology (PR = 1.55, 95% CI: 1.31–1.85, *p* < 0.001). The table also reports results for other factors, such as age, which showed a significant association with depressive symptomatology (PR = 1.03, 95% CI: 1.01–1.06, *p* = 0.012) and anxiety symptomatology (PR = 1.09, 95% CI: 1.04–1.13, *p* < 0.001). Resilience displayed a significant association with depressive symptomatology (PR = 0.99, 95% CI: 0.99–1.00, *p* = 0.056) and anxiety symptomatology (PR = 0.99, 95% CI: 0.98–0.99, *p* = 0.012).

Furthermore, the Family APGAR score had different levels of association with mental health outcomes, with mild, moderate, and severe categories exhibiting significant associations with depressive and anxiety symptomatology compared to the normal category. Other factors, such as sex, alcohol consumption, and confidence in government to manage the COVID-19 epidemic, exhibited varying levels of association with the outcomes.

## Discussion

The global outbreak of the COVID-19 pandemic has presented unparalleled difficulties to individuals, families, and communities worldwide. The prolonged and uncertain nature of the pandemic has disrupted the lives of adolescents in numerous ways, impacting their physical, emotional, and mental well-being. In this study, we aimed to investigate the prevalence of depressive and anxiety symptomatology among adolescents in the context of the COVID-19 pandemic, as well as identify factors associated with these mental health issues.

Our study’s research framework, illustrated in Figure 1, demonstrates the complex interplay of factors contributing to the mental health outcomes of schoolchildren during the COVID-19 pandemic. The significant associations between childhood trauma, depressive symptomatology, and anxiety symptomatology emphasize the importance of addressing and mitigating the effects of trauma in this population.

### Prevalence of depressive and anxiety symptomatology

Our findings indicate a high prevalence of depressive and anxiety symptomatology among adolescents during the COVID-19 pandemic. Specifically, 7 out of 10 adolescents reported symptoms of depression, with 12.9% experiencing severe symptomatology. Meanwhile, 6 out of 10 adolescents reported symptoms of anxiety, with 14.5% presenting severe symptomatology. Our data suggest a higher prevalence of depressive and anxiety symptomatology during the pandemic than reported in previous studies in Chile and the United States (69, 70). This increase could be due to the restrictions and social isolation caused by the COVID-19 pandemic, as adolescents were less motivated to participate in activities they normally enjoyed, such as physical activity (71). Additionally, the risk of exposure to domestic violence (72), child abuse (73), and the acute and persistent impact of COVID-19 (74) may have increased depressive and anxious symptoms in adolescents (11, 75).

### Association between childhood trauma and mental health

In our study, we found a strong association between childhood trauma and mental health issues such as depressive and anxiety symptomatology in adolescents. This finding aligns with previous research, which has consistently shown that traumatic experiences during childhood can act as potent risk factors for the onset, symptomatic severity, and course of depression and anxiety disorders (76). Our results contribute to the growing body of evidence highlighting the critical need to address childhood trauma and its long-lasting consequences on mental health and overall well-being.

The relationship between childhood trauma and mental health issues can be understood through various theoretical frameworks. One such framework is the stress-diathesis model, which suggests that an individual’s vulnerability to developing mental health disorders is determined by a combination of genetic predispositions and environmental stressors (77, 78). Childhood trauma may act as a significant environmental stressor, triggering the onset of mental health issues in individuals with a genetic predisposition.

Furthermore, childhood trauma can lead to maladaptive coping strategies and cognitive distortions, such as negative self-appraisal, a sense of helplessness, and difficulties in emotion regulation (79). These maladaptive patterns may persist into adolescence and adulthood, increasing the risk of developing depression and anxiety disorders. In addition, early traumatic experiences can disrupt the normal development of neural circuits, leading to alterations in brain structure and function that predispose individuals to mental health issues (79).

Our findings emphasize the importance of early intervention and support for children exposed to traumatic experiences. Interventions such as trauma-focused cognitive-behavioral therapy (TF-CBT), eye movement desensitization and reprocessing (EMDR), and family-based interventions have shown promise in mitigating the adverse effects of childhood trauma on mental health (80–82). These therapeutic approaches aim to address trauma-related cognitive distortions, improve emotion regulation skills, and foster a supportive environment for healing.

### Other factors associated with depressive symptoms

In addition to childhood trauma, our study identified several other factors associated with depressive symptoms among adolescents during the COVID-19 pandemic. For each additional year of age, the prevalence of depressive symptoms slightly increased. This may be since children and adolescents are in constant development, and they are particularly vulnerable to social exclusion, discrimination, educational difficulties, negative interpersonal relationships, and environmental factors that can affect their mental health (83). Additionally, adolescents who reported seeking mental health help had a higher prevalence of depressive symptomatology. This could be due to the stigma and negative beliefs that may exist toward mental health services and professionals (84). Therefore, it is essential to adopt effective intervention strategies to address adolescent mental health, such as self-directed cognitive-behavioral interventions that can be implemented in various settings, including schools, communities, health centers, and camps (85, 86).

### Other factors associated with anxiety symptoms

Like depressive symptoms, several factors were associated with anxiety symptomatology among adolescents during the COVID-19 pandemic. For each additional year of age, the prevalence of anxiety symptoms slightly increased. This is consistent with previous studies that have found anxiety tends to increase with age and can persist into adulthood (87, 88). Adolescents who perceived the pandemic’s severity neutrally had a lower prevalence of anxiety symptoms. This suggests that providing social support and mindfulness strategies to help adolescents cope with the pandemic can reduce anxiety symptoms (89). Additionally, adolescents from dysfunctional families had a higher prevalence of anxiety symptomatology. Family dysfunction can create conditions for adolescents to experience a greater sense of hopelessness, changes in their lifestyle, and mental health problems, with anxiety being the most prevalent (90). Finally, our study found that resilience was negatively associated with anxiety symptoms. Adolescents with higher resilience scores experienced lower prevalence of anxiety symptoms, suggesting that promoting resilience can have positive effects on adolescent mental health during the pandemic (91).

### Limitations and strengths

Our study has limitations that must be acknowledged. First, the cross-sectional design cannot establish causality. Second, nonresponse bias may have affected the results, as levels of adolescents’ motivation for voluntary participation in the study may have varied. Third, self-reported data could be subject to information bias. Fourth, certain variables, such as eating disorders (92), personality disorder (93), and post-traumatic stress disorder (94), could not be measured due to the secondary data analysis (95). Lastly, selection bias may be present, as all study subjects voluntarily participated in the questionnaire. However, our study has notable strengths. Firstly, we used a validated and reliable questionnaire to assess childhood trauma and mental health problems among adolescents. Secondly, we obtained a sufficient sample size to achieve adequate statistical power and reduce the risk of type II error. Thirdly, we used the snowball method, which is a cost-effective and convenient way to recruit study participants. Fourthly, we conducted the study during the second wave of the COVID-19 pandemic, which allowed us to capture the effects of a prolonged period of quarantine and social isolation on mental health outcomes. Lastly, our findings provide valuable insights into the association between childhood trauma and mental health problems in the context of the COVID-19 pandemic, which can inform the development of targeted interventions to support vulnerable adolescents.

### Relevance of findings in mental health

The COVID-19 pandemic has profoundly impacted the mental health of young people, posing urgent and potentially long-term challenges (96). To control the pandemic, global measures such as mass quarantine, confinement, social distancing, and school closures have been implemented, drastically altering the lives of children and adolescents. As a result, they have had to endure extended periods of isolation and restricted social interactions (97). These unprecedented circumstances have not only led to changes in adolescent behavior but also exacerbated mental health disorders, particularly for those with previous experiences of childhood trauma (98). While the adverse effects of the pandemic on adolescent health and well-being have lessened, mental health problems have seen a significant increase (99). Studies report a high prevalence of mental health conditions (83%), including depression (29%), anxiety (26%), sleep disorders (44%), and post-traumatic stress symptoms (48%) (12, 23, 100).

In the context of the COVID-19 pandemic, childhood trauma has been identified as a predictor of poor mental health outcomes, partly due to the increased psychological impact associated with the pandemic (e.g., intrusion, hyperarousal, avoidance, depressive symptoms, anxiety symptoms, and stress symptoms) (29). Consequently, it is crucial to monitor the mental health repercussions of the pandemic on adolescents, particularly those from vulnerable backgrounds. Factors that may exacerbate vulnerability include lower socioeconomic status, parents with higher levels of psychopathology, increased familial conflict, irregular routines, prior psychiatric diagnoses, and greater exposure to COVID-19 (101).

The results of the study can help schools in several practical ways. Firstly, the study found that childhood trauma was strongly associated with depression and anxiety in schoolchildren. Therefore, schools can use this information to identify students who have experienced childhood trauma and provide them with appropriate support and interventions to prevent the development of mental health disorders.

Secondly, the study found that previous history of mental health disorders was strongly associated with depression and anxiety. Schools can use this information to identify students who have a history of mental health disorders and provide them with appropriate support and interventions to prevent relapse or further deterioration of their mental health.

Thirdly, the study found that resilience was protective against depression and anxiety. Schools can use this information to implement programs and activities that promote resilience among their students, such as mindfulness training, social and emotional learning, and physical exercise.

By understanding and addressing these factors, we can better support the mental health and well-being of adolescents during and beyond the pandemic.

## Conclusion

In conclusion, this study sheds light on the significant impact of the COVID-19 pandemic on adolescent mental health, particularly concerning depressive and anxiety symptoms. Our findings reveal a strong association between childhood trauma and mental health issues, emphasizing the importance of early intervention and support for affected adolescents. The pandemic has exacerbated pre-existing vulnerabilities, underscoring the need to closely monitor and address mental health challenges among at-risk adolescents.

Our findings hold clinical utility and can inform healthcare providers, policymakers, and educators about the importance of addressing the mental health needs of adolescents who have experienced childhood trauma, especially during challenging times such as the pandemic. This knowledge may be applied in developing school-based interventions aimed at preventing or treating depression and anxiety in students who have experienced childhood trauma and have been exposed to the COVID-19 pandemic. For example, schools could provide targeted mental health support to this vulnerable group, such as access to counseling services or group therapy sessions.

Additionally, our study highlights the importance of addressing childhood trauma as a risk factor for mental health problems, particularly during times of increased stress and uncertainty. Future research should build on this study by focusing on the originality and novelty of such interventions, exploring innovative approaches, utilizing cutting-edge technology, or fostering interdisciplinary collaborations to better understand the complex interplay of factors contributing to poor mental health outcomes. Longitudinal studies investigating the long-term effects of the pandemic on adolescent mental health and resilience would also provide invaluable insights for designing targeted, evidence-based interventions. By committing to these research endeavors, we can ensure that essential resources and support are available to foster resilience and recovery in the face of adversity, ultimately improving the well-being of adolescents during and beyond these unprecedented times.

## Data availability statement

The data analyzed in this study is subject to the following licenses/restrictions: The ethics committee restricts the public use of the dataset. However, they are available from authors on reasonable request. Requests to access these datasets should be directed to MV-G, josvg44@gmail.com.

## Ethics statement

The studies involving human participants were reviewed and approved by the Ethics and Research Committee of the Universidad San Martín de Porres, Lima, Peru. Written informed consent to participate in this study was provided by the participants’ legal guardian/next of kin.

## Author contributions

MV-G, DL-F, FD, SB-C, MF-C and DV-G conceived and designed the study, collected and analyzed the data, and wrote the manuscript. MV-G, SB-C, MF-C, VF-R, and CP-V contributed to the analysis and interpretation of the data and critically revised the manuscript for important intellectual content. MV-G, VF-R DV-G, CP-V and FI-B provided valuable input and contributed to the writing and revision of the manuscript. MV-G, DL-F, and VF-R take responsibility for the integrity of the work as a whole, from inception to publication. All authors have read and approved the final version of the manuscript.

## Conflict of interest

The authors declare that the research was conducted in the absence of any commercial or financial relationships that could be construed as a potential conflict of interest.

## Publisher’s note

All claims expressed in this article are solely those of the authors and do not necessarily represent those of their affiliated organizations, or those of the publisher, the editors and the reviewers. Any product that may be evaluated in this article, or claim that may be made by its manufacturer, is not guaranteed or endorsed by the publisher.
